# Morphology, Volume, and Density Characteristics of the Parotid Glands before and after Chemoradiation Therapy in Patients with Head and Neck Tumors

**DOI:** 10.1155/2020/8176260

**Published:** 2020-03-26

**Authors:** Wellington Pereira dos Santos, João Pedro Perez Gomes, Amanda Drumstas Nussi, Joa˜o Mauricio Altemani, Maria Teresa Botti Rodrigues dos Santos, Bengt Hasseus, Daniel Giglio, Paulo Henrique Braz-Silva, Andre Luiz Ferreira Costa

**Affiliations:** ^1^Faculty of Medical Sciences, University of Campinas (UNICAMP), Campinas, SP, Brazil; ^2^Department of Stomatology, School of Dentistry, University of Sa˜o Paulo (USP), Sa˜o Paulo, SP, Brazil; ^3^Postgraduate Program in Dentistry, Cruzeiro do Sul University (UNICSUL), Sa˜o Paulo, SP, Brazil; ^4^Department of Radiology, Faculty of Medical Sciences, University of Campinas (UNICAMP), Campinas, SP, Brazil; ^5^Department of Oral Medicine and Pathology, Institute of Odontology, The Sahlgrenska Academy, University of Gothenburg, Gothenburg, Sweden; ^6^Department of Oncology, Institute of Clinical Sciences, University of Gothenburg Sahlgrenska Academy, Gothenburg, Sweden; ^7^Laboratory of Virology, Institute of Tropical Medicine of São Paulo, School of Medicine, University of São Paulo (USP), São Paulo, SP, Brazil

## Abstract

The multimodal approach for patients with head and neck cancer (HNC) includes treatment with chemoradiation therapy (CRT). A common concern regarding CRT side effects is the occurrence of structural and physiological alterations of the salivary glands due to exposure to ionizing radiation. The aim of this study is to examine the morphology, volume, and density of the parotid glands before and after CRT in HNC patients. A total of 49 HNC patients treated exclusively with CRT were included in the study. Ninety-eight parotid glands were evaluated before and after treatment by using contrast-enhanced computed tomography (CECT). Shapiro–Wilk test was performed, and the variables (pre-CRT and post-CRT) presented normal distribution. Pearson's coefficient was used to assess the correlation between volume and density. CRT resulted in a significant decrease in the mean volume of the parotid glands (i.e., original volume reduced by 20.5%; *P* < 0.0001). CRT induced a 30.0% (7 Hounsfield units) increase in density of the right parotid gland and a 24.9% (8 Hounsfield units) increase in density of the left parotid gland (*P*=0.0198 and *P*=0.0079, respectively). Changes in morphology and spatial configuration, increased density, and substantial loss of volume of the parotid glands were observed after CRT. There was also a difference in density (*P*=0.003) in the right-side parotid glands in comparison between xerostomic and nonxerostomic groups of patients. These facts lead to the need for a personalized CRT planning in order to minimize oral complications related to the treatment.

## 1. Introduction

Head and neck cancer (HNC) treatment requires a multidisciplinary approach, which may include surgical resection, radiation therapy (RT), and chemotherapy. Studies have demonstrated that concurrent chemoradiotherapy (CRT) is required for locally advanced HNC. Head and neck squamous cell carcinoma (HNSCC) is a heterogeneous disease encompassing lesions that originate in the oral cavity, lips, oropharynx, hypopharynx, nasopharynx, or larynx. Worldwide, HNSCC represents the sixth most common neoplasia and constitutes more than 90% of HNC cases [1].

Considering that RT has long been the mainstay of treatment for HNC patients, traditionally involving a stage-dependent strategy whereby all patients with the same TNM stage receive the same therapy, the authors have proposed approaches aiming at personalized guidance and biological adaptation of RT [2, 3].

Over the last years, significant progress in image-guided RT has allowed identifying significant volumetric changes in organs at risk (OAR), which can occur during a typical course of intensity-modulated radiation therapy (IMRT) for HNC patients. In addition, the literature has also emphasized that many medical centers carry out the original RT plan for HNC patients without considering anatomical changes that can occur due to weight loss, edema, inflammation, tumor shrinkage, and changes in normal tissue volume. Thus, volumetric changes can have a profound impact on the delivered doses to the tumor and normal tissues [4]. The volume of normal tissues being irradiated is determined by the size of the target and RT technique [5].

RT has been driven by technological advances and approximately 50% of all patients with localized malignant tumors are treated with RT at some point in the course of their disease [6]. RT is used to treat approximately 80% of HNC patients [5]. However, despite all the technological advances, it is a key challenge to maximize the radiation doses to cancer cells and at the same time to minimize damage to the surrounding healthy tissues [7]. RT destroys cancer cells by depositing high physical energy to the cells [6] and at the same time causing numerous epithelial and stromal changes (e.g., fibrosis) in the normal tissue surrounding the tumor leading to early and late adverse effects RT [8]. The radiation dose, the localization of the tumor, and the sensitivity of the normal tissue next to the tumor are all factors affecting the severity of adverse effects to RT [5]. RT may cause xerostomia, dysphagia, mandibular osteoradionecrosis, trismus, hearing loss, and radiation caries [5, 9].

Studies have compared the levels of incidence of moderate to severe late xerostomia in two-dimensional (2D) RT and IMRT. In 2D-RT, the incidence of xerostomia varied from 60% to 75%, whereas the incidence was around 40% for IMRT [5]. Radiation-induced xerostomia is caused by a reduced volume of saliva secretion (gland hypofunction) or change in salivary composition, which may affect swallowing and speech, besides compromising oral health [5]. Xerostomia is the most common radiation-induced complication in HNC patients, which can significantly reduce the quality of life [10–13].

Considering that salivary glands are usually irradiated during RT for HNC, which leads to radiation-induced xerostomia [10], the aim of this study was to assess the morphology, volume, and density changes in parotid glands induced by CRT in patients with HNSCC with the aid of contrast-enhanced computed tomography (CECT).

## 2. Materials and Methods

### 2.1. Patients

The present retrospective study was submitted to and authorized by the Research Ethics Committee of the University of Campinas according to protocol number 79765917.5.0000.5404. All procedures performed in the study were in accordance with human research ethical standards set by institutional and/or national research committees and with the 1964 Helsinki Declaration, including later amendments or comparable ethical standards. A written informed consent form was signed by all patients and relatives of the deceased ones. Stages were defined according to criteria established by the American Joint Committee on Cancer (AJCC). All patients underwent CECT of the head and neck before and after CRT.

### 2.2. Radiation Therapy

All the 49 patients were treated with radiotherapy by using conventional 2D technique, in which two lateral bundles are attached to the face and the upper neck, plus an anterior bundle aimed to cover the lower neck. A total dose of 7,000 centigray (cGy) was given, divided into 35 fractions of 200 cGy each. RT sessions were performed once a day for five days a week until completing the entire cycle (7000 cGy).

### 2.3. Concurrent Chemotherapy

The patients received intravenous cisplatin (100 mg/m^2^), administrated on days 1, 22, and 43 after the start of RT (day 1). As for the antiemetic and hydration protocols, all patients received intravenously 3,000 ml of normal saline solution (0.9%), 125 ml of mannitol 20%, 24 mg of ondansetron, and 20 mg of dexamethasone before cisplatin infusion, as well as 2,000 ml of saline solution 0.9% (also intravenously) and oral administration of 8 mg of dexamethasone (every 12 hours) and 10 mg of metoclopramide (every 6 hours) during three days after each infusion [14, 15].

### 2.4. Image Acquisition

CECT scan was performed by using a 64-channel Computed Tomography scanner (Aquilion model, Toshiba Medical Systems Corporation) with multislice system and field of vision (FOV) of 320 mm, yielding slice thickness and reconstruction interval of 3 mm each. The scanner operated at 120 kVp and 400 mA.

### 2.5. Image Processing and Analysis

Image processing, visualization, volume, and density measurements were performed on a 6-inch thick Lenovo notebook with Intel core i7 64-bit processor, on-board video card, 15-inch monitor and running under Windows 10® platform (Microsoft Corp., USA). A single radiologist performed the delineation of all parotid glands (i.e., segmentation process). An example of lesion scans (not from the study sample) was shown to the examiner before the beginning of the segmentation process. After the examiner's calibration, the segmentation was performed twice for all parotid glands in order to ensure that the volume and density data were not distorted by poor estimation.

**Figure 1:**
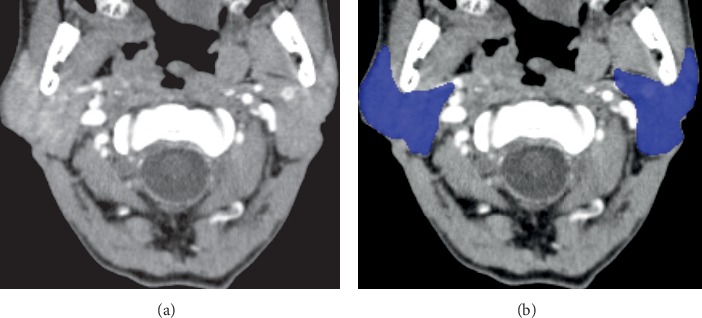
Enhanced-contrast computed tomography (CECT) showing parotid glands structures before chemoradiation therapy (CRT) in the axial slice (a). CECT showing the parotid glands delineation after the segmentation process with the aid of computer software (b).

The segmentation process was performed with the aid of the software InVesalius, version 3.1 (CTI, Campinas, Brazil), which is freely available on http://www.cti.gov.br/invesalius. Segmentation is an important parameter for the generation of three-dimensional (3D) reconstruction models, as it allows the evaluation of density, volume, and morphology of the target structures, such as the parotid glands (Figure 1(a)).

For this, the proper threshold was determined to separate the structures of interest from the surrounding structures. This tool is based on a gray-level range that expresses only the pixels corresponding to the structures to be studied, in this case, the parotid glands. When this interval is inadequately determined, the structures to be analyzed may be thinned, thickened, or even suppressed from the reconstruction process, leading to possible misinterpretation. In this study, the parotid threshold was determined empirically. The precise delineation of the gland was made possible by using intravenous contrast (i.e., 612 mg/mL of iopamidol—an iodine nonionic contrast agent), which was administrated prior to the computed tomography (CT) scan. Through an accurate observation, it was possible to manually determine the gland boundaries in all CECT slices. The segmented structures were divided and highlighted with different colors (Figure 1(b)).

The segmentation process was manually performed at two time points before and after CRT. Through the segmentation of the parotid glands, a 3D reconstruction model was properly analyzed for volume, density, spatial, and morphological changes. The volume of the glands was calculated and expressed in cubic millimeters (mm^3^). The density was measured in Hounsfield unit (HU) scale. In addition, morphological and spatial alterations were analyzed by assessing the anatomical characteristics of the gland on a 3D virtual model. The follow-up period of the control CECT (post-CRT treatment) ranged between three and 24 months (median of 5 months).

### 2.6. Critical Data Analysis

Exploratory data analysis was performed by using summary measures (i.e., mean, standard deviation, minimum, median, maximum, frequency, and percentage). Shapiro–Wilk test was performed and normal distribution of the data was confirmed. Volume and density of the parotid glands were compared before and after CRT by using paired Student's *t*-test, whereas genders were compared regarding the variation of the variables of interest by using two-tailed Student's *t*-test. Comparison of volume and density variations between xerostomic and nonxerostomic groups were performed using Student's *t*-test, and tumor stage and location by using analysis of variance (ANOVA). The correlations of age and interval with the variation of the three variables of interest were assessed by using Pearson's correlation coefficient. Pearson's correlation coefficient was also used to assess the correlation between volume and density.

The resulting data were submitted to statistical analysis by using the software SAS System for Windows (Statistical Analysis System), version 9.4 (SAS Institute Inc, Cary, NC, USA) and R version 3.4.2. (Copyright© 2017 The R Foundation for Statistical Computing, Vienna, Austria).

## 3. Results

### 3.1. Clinical Aspects of the Patients

The demographics, details of cancer disease, and CECTs of the 49 patients with HNSCC in the study are presented in Table 1. Of these 49 patients, 45 were males and four were females. The majority of the patients were Caucasian (*n* = 42). Tumors were situated in the oropharynx (*n* = 27), followed by the oral cavity (*n* = 11), larynx (*n* = 7), and other sites (*n* = 4). Patients with advanced stages (IVa and IVb) consisted the majority of the cohort (82%).

### 3.2. Morphological Changes and Spatial Migration

Visual comparison of CECT images prior and post-CRT showed important morphological changes. After treatment, the majority of the analyzed parotid glands lost its convexity and tended to a flat or concave shape, suggesting that one of the immediate effects of CRT involves the shrinkage of the salivary glands. Regarding spatial position, the CRT resulted in a migration of the parotids toward the midline and superior directions.

### 3.3. Volume and Density Analysis before and after CRT

**Figure 2:**
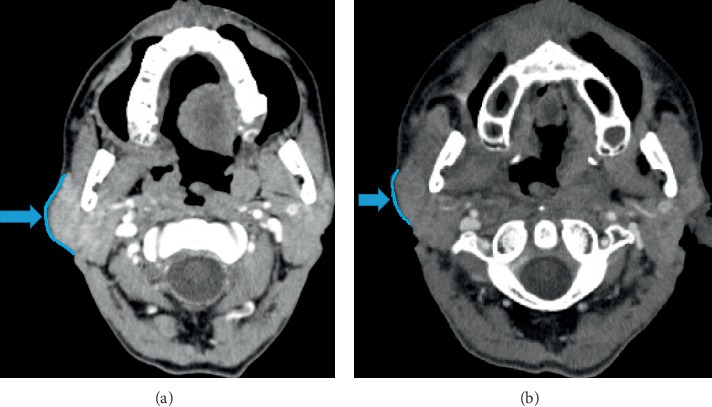
Enhanced-contrast computed tomography (CECT) showing the convexity of the parotid gland prior to chemoradiation therapy (CRT) (a). CECT showing the tendency of the parotid gland to flatten in the post-CRT period (b). The CRT resulted in a migration of the parotids toward the midline (medial) and superior (cranial) directions. This migration can be explained by the fact that the lateral boundaries of the gland suffer more directly by the effects of radiation, considering it is not surrounded by bone structures. Therefore, the gland loses its convexity and tends to acquire a flat or even concave shape.

A significant decrease by 13.124 mm^3^ of the mean total parotid volume occurred in patients undergoing CRT, corresponding to 20.5% of the original volume (*P* < 0.0001). The left and right parotid glands were affected similarly by CRT; i.e., the volumes were reduced by 21% (6.809 mm^3^) and by 20% (6.318 mm^3^), respectively (*P* < 0.0001). CRT also affected the density of the parotid glands; i.e., the mean density increased by 7 HU (30.0% increase) and by 8 HU (24.9%) for the right and left parotids, respectively (*P*=0.0198 and 0.0079, respectively; Table 2). Morphological, volume, and density changes in CECT images of parotid glands pre- and post-CRT are illustrated in Figures 2(a) and 2(b).

**Figure 3:**
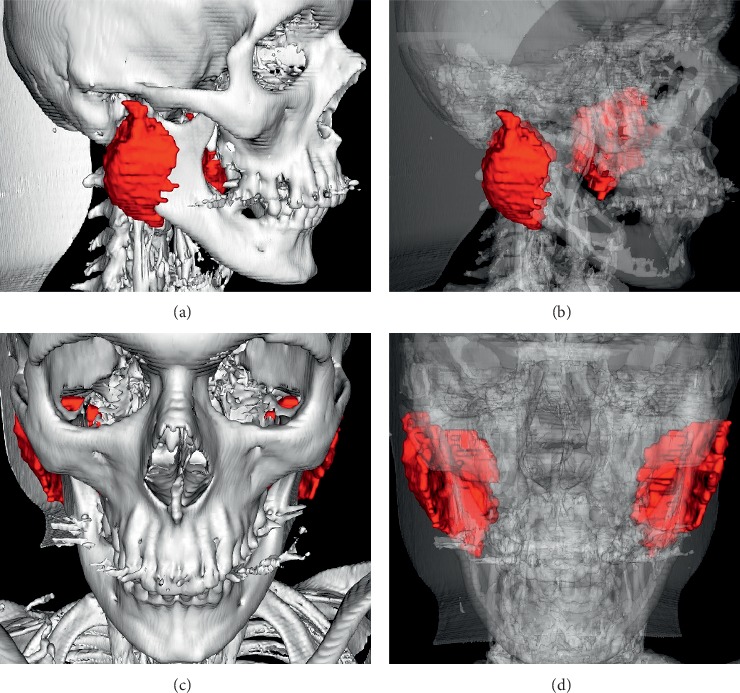
Lateral view of the skeleton after automatic segmentation based on preestablished thresholds (minimum: 226/maximum: 3071) along with parotid glands before chemoradiation therapy (CRT) (a). Lateral view of the skeleton with reduced bone transparency aiming to visualize the morphology of the parotid gland and perform volumetric analysis (b). Frontal view of the skeleton along with parotid glands prior to CRT (c). Frontal view of the skeleton with reduced bone transparency prior to CRT (d).

**Figure 4:**
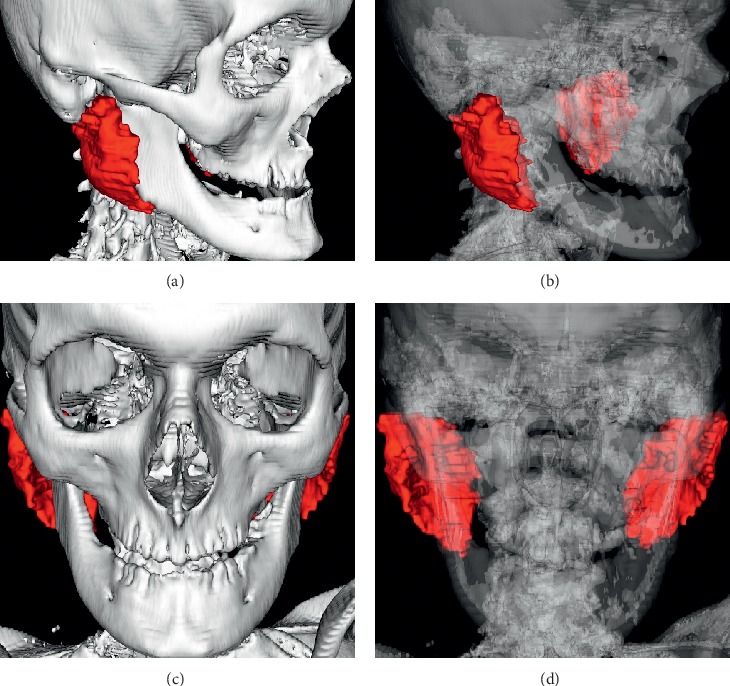
Lateral view of the skeleton after automatic segmentation along with parotid glands in the postchemoradiation therapy (CRT) period. Lateral view of the skeleton with reduced bone transparency aiming to visualize the morphology of the parotid gland and perform volumetric analysis (b). Our results show that CRT may result in a significant decrease in the mean parotid volume. Frontal view of the skeleton along with parotid glands in the post-CRT period (c). Frontal view of the skeleton with reduced bone transparency in the post-CRT period (d). The morphological alterations seen in this study can be associated with a significant decrease in the volume of the parotid gland together with a displacement of its center of mass toward medial and cranial directions. This implies substantial alterations in the lateral and inferior surfaces of the gland.

The 3D representation model of the parotid glands prior to CRT is presented in Figures 3(a)–3(d). The 3D representation model of the parotid glands post-CRT is presented in Figures 4(a)–4(d).

### 3.4. Volume and Density Comparisons between Genders

The comparison between genders for volume and density variations in the parotid gland is presented in Table 3. This table shows a greater variation in the volume reduction after CRT among male patients for three variables: total volume, right-side volume, and left side volume (*P* value = 0.001, 0.008, and <0.001, respectively). With regard to density variation, no significant difference was found between genders. However, it is important to highlight that there is a substantially higher number of males in our sample (*n* = 45) in comparison to females (*n* = 4).

### 3.5. Volume and Density versus Tumor Location and Stage

Volume and density values of parotid glands were obtained to evaluate possible variations between tumor location and stages. The anatomical sites comprised oropharynx (*n* = 27), oral cavity (*n* = 11), larynx (*n* = 7), hypopharynx (*n* = 3), and rhinopharynx (*n* = 1). No significant differences were found between tumor location with regard to volume and density variations in parotid glands. Meaning that according to this sample analysis, the head and neck sites where the neoplasm arises are not a factor responsible for causing density or volumetric changes in parotids. Similarly, no significant differences were observed between the groups of stages in relation to the assessed variables.

### 3.6. Volume and Density versus Xerostomia

Patients were divided into xerostomic (*n* = 42) and nonxerostomic (*n* = 5) groups. In two cases of the sample, xerostomia could not be assessed due to the absence of this information in medical records. Both groups were analyzed in order to assess possible variations regarding tumor volume and density and its association with xerostomia condition. The nonxerostomic group presented a reduced density on the right-side parotids, whereas xerostomic group presented an increased density (*P* value = 0.003).

## 4. Discussion

RT is considered crucial for the treatment of HNC, especially in those cases where surgery is impracticable. In fact, with regard to early stage oropharyngeal squamous cell carcinoma (OPSCC), the longer follow-up did not show significant differences in overall survival and locoregional recurrence-free survival (LRFS) between these two modalities of treatment [16]. However, considering that RT side effects may include grade 3 toxicity, grade 4 osteoradionecrosis of the jaw, and radiation-induced xerostomia, the effects of RT during HNC treatment are not negligible [10, 16]. In fact, our study shows that a series of alterations in parotid glands occurred during CRT, including morphological, volumetric, spatial, and density-related ones. The degree of severity regarding salivary gland damage during CRT can be associated with the chosen technique as conventional 2D-RT may worsen the side effects compared to IMRT [5].

The morphological alterations seen in this study occurred due to a significant decrease of the parotid volume, with the displacement of its center of mass toward the medial and cranial directions. This implies significant alterations in the lateral and inferior surfaces of the gland. The deformation of the glands seen in this work is in accordance with previous studies [10, 17, 18].

The parotid shrinkage seen in this study led to a shift from a convex to a concave surface during RT, similarly to that reported by Juan et al. [19]. It was also shown that this gland tends to reassume its convex shape within 435 days after completion of treatment [19]. We have hypothesized that high-dose radiation to the parotid glands could destroy acini and granules, which would result in changes in blood velocity. Moreover, the scant information about the relationships between these parameters and the radiation dose delivered to the glands emphasizes the necessity of further studies. In fact, the knowledge of these relationships can provide useful references for prediction of the severity of xerostomia after RT, and consequently, a better understanding of how this complication will affect the quality of life in the long-term survivor [20].

Our results have shown a volume reduction of 21% in the parotid glands on both sides, which is similar to the results presented by Cheng et al. [10] and 15% lower than the results reported by Wu et al. [20]. These variations in volume may be associated with several causes, including radiation dosage, interval of sections, gender (a majority were males in our cohort), tumor location, and RT technique. We believe that volumetric changes are also related to a greater or lesser degree of tissue vascularization. Patients with more vascularized parotid glands have a greater volume reduction, possibly due to increased tissue oxygenation, which leads to higher radiosensitivity of the gland [21]. Moreover, the reduced volume of parotid glands might be due to acinar loss or fibrosis [19].

With regard to density changes, a significant variation was found in our study. In fact, increases of 30% on the right parotid gland and 24.9% on the left one (measured in HU) were observed, which corroborates the findings by Cheng et al. [10], Ogura et al. [11], and Wu et al. [20]. However, Fiorino et al. [22] and Xu et al. [23] found reductions in the density of the glands. This discrepancy can be explained by differences in the radiation dosage. Other studies [10, 11] demonstrated that an increased density of salivary glands was observed with doses above 4.500 cGy, which was the case with all the patients in our study. Moreover, studies reporting a decrease in the density values point to a possible association with the gland's deformation [22].

With regard to density changes and xerostomia condition, our results showed an increased density on the right-side parotid glands in xerostomic groups, whereas a reduced density was observed in nonxerostomic groups after treatment. These findings are in concordance with Pinna et al. [24], since a preventive approach and a personalized CRT is of extreme importance to prevent and improve the xerostomia condition.

It is interesting to assess alterations in the volume and density of the parotid glands because this allows for a personalized treatment plan based on the prediction of changes the salivary glands will suffer during CRT. These analyses also allow identifying patients with higher or lower radiosensitivity patterns, which enables us to plan the post-CRT care and to perform a personalized treatment. This approach is essential to ensure that the tumor site receives proper radiation doses while preventing other organs at risk (e.g., parotid glands) from receiving radiation doses above their tolerance. It is important to highlight that acinar atrophy and chronic inflammation of the salivary glands are hallmarks of RT [25], and because the management of xerostomia is rarely effective, prevention is paramount [26]. However, these preventive approaches are not applicable to all patients, which emphasizes the need for personalized treatments and also further studies incorporating new biological insights in order to optimize the therapeutic index of RT for HNC [26].

## 5. Conclusion

Chemoradiation therapy has severe implications in the morphology of the parotid glands, with volume loss and density increase that could explain salivary gland hypofunction and xerostomia in head and neck cancer patients. A preventive approach and personalized CRT treatment can help to prevent and to manage xerostomia effects, thus increasing the quality of life of oncological patients.

## Figures and Tables

**Table 1:** Patients description (age in years and the interval between CECTs in months).

Variable	Mean ± SD	Median	Min-max	*N*	Percentage (%)
Age	59.6 ± 9.9	59	42–89		
Interval	7.1 ± 5.4	5	2–28		

*Gender*					
Male				45	91.8
Female				4	8.2
Total				49	100

*Tumor location*					
Oral cavity				11	22.5
Hypopharynx				3	6.1
Larynx				7	14.3
Oropharynx				27	55.1
Rhinopharynx				1	2
Total				49	100

*Skin color*					
White				42	85.7
Not-white				7	14.3
Total				49	100

*Stage*					
I				1	2
III				8	16.3
IVa				34	69.4
IVb				6	12.2
Total				49	100

CECT: contrast-enhanced computed tomography; *N*: sample size; SD: standard deviation; min: minimum; max: maximum.

**Table 2:** Statistic measures of pre- and post-RT volume and density, variation, and percentage of variation and time comparison (Student's *t*-test).

Variable	Time	*N*	Mean	SD	Min	Median	Max	*P* value
Total volume	Pre-RT	49	58819	18125	26127	56879	104603	**<0.0001**
	Post-RT	49	45695	13844	22847	47039	81123	
	Variation	49	−13124	11576	−45630	−9675	9845	
	Variation %	49	−20.5	16.2	−60.2	−19.2	21.7	

Right parotid volume	Pre-RT	49	29274	9499	11366	28727	55220	**<0.0001**
	Post-RT	49	22465	6687	11476	22358	39003	
	Variation	49	−6809	5871	−21744	−5539	6121	
	Variation %	49	−21.0	16.4	−59.3	−19.3	28.5	

Left parotid volume	Pre-RT	49	28477	8923	14445	28049	49541	**<0.0001**
	Post-RT	49	23159	7251	11304	23183	42119	
	Variation	49	−6318	6245	−23883	−4488	6033	
	Variation %	49	−19.8	−17.4	−61.1	−18.6	21.1	

Right parotid density	Pre-RT	44	59	22	11	60	121	**0.0198**
	Post-RT	44	67	19	27	67	109	
	Variation	44	7	20	−45	6	50	
	Variation %	44	30.0	79.5	−48.5	9.7	455.7	

Left parotid density	Pre-RT	44	59	21	13	59	102	**0.0079**
	Post-RT	44	67	21	17	66	103	
	Variation	44	8	19	−48	7	46	
	Variation %	44	24.9	62.0	−47.3	9.8	350.7	

RT: radiation therapy; *N*: sample size; SD: standard variation; min: minimum; max: maximum. Regarding density variables, 5 patients were disregarded for being considered outliers. Volume (mm^3^).

**Table 3:** Comparison of variation in volume and density between genders (Student *t*-test).

Variable	Gender	*N*	Mean	SD	Min	Median	Max	*P* value
Total volume	Female	4	−3786	3082	−6964	−4297	415	0.001
	Male	45	−13954	11701	−45630	−11932	9845	

Right parotid volume	Female	4	−2596	2055	−4910	−2784	92	**0.008**
	Male	45	−7184	5963	−21744	−7484	6121	

Left parotid volume	Female	4	−1316	1249	−2565	−1513	325	**<0.001**
	Male	45	−6762	6322	−23883	−5179	6033	

Right parotid density	Female	4	1.6	8.6	−11.1	4.7	7.93	**0.273**
	Male	40	8.1	21.2	−45.4	5.9	50.2	

Left parotid density	Female	4	9.4	4.6	4.0	9.6	14.33	**0.674**
	Male	40	7.7	19.6	−48.4	6.9	45.9	

*N*: sample size; SD: standard deviation; min: minimum; max: maximum. Volume (mm^3^).

## Data Availability

The data used to support the findings of this study are available to interested readers upon reasonable request.
